# (2,2′-Bipyridine)(2-{1-[2-(dimethyl­amino)ethyl­imino]eth­yl}-4-methoxy­phenolato)copper(II) perchlorate

**DOI:** 10.1107/S1600536809014573

**Published:** 2009-04-30

**Authors:** Yueh-Hsuan Tsai, Wen-Chou Hung, Chu-Chieh Lin

**Affiliations:** a Department of Chemistry, National Chung Hsing University, Taichung 402, Taiwan, Republic of China

## Abstract

The Cu atom of the title complex, [Cu(C_13_H_19_N_2_O_2_)(C_10_H_8_N_2_)]ClO_4_, has a distorted square-pyramidal geometry with all three of the donor atoms from the *N*,*N*′,*O*-tridentate Schiff base ligand in the equatorial positions and the bipyridine N atoms in an equatorial–axial binding mode. The Cu atom is 0.1801 (11) Å above the N_3_O mean basal plane.

## Related literature

For the development of efficient catalytic systems for the coupling of CO_2_ with heterocycles into polycarbonates, see: Inoue *et al.* (1969 ▶). For the synthesis and catalytic studies of a series of *bis­*–(salicylaldiminato)zinc complexes, see: Darensbourg *et al.* (2001 ▶). For similar complexes, see: Dhar *et al.* (2006 ▶); Shen *et al.* (2003 ▶). For the synthesis, see: Hung & Lin (2009 ▶); Hung *et al.* (2008 ▶); For the chemical activity of complexes, see: Noh *et al.* (2007 ▶).
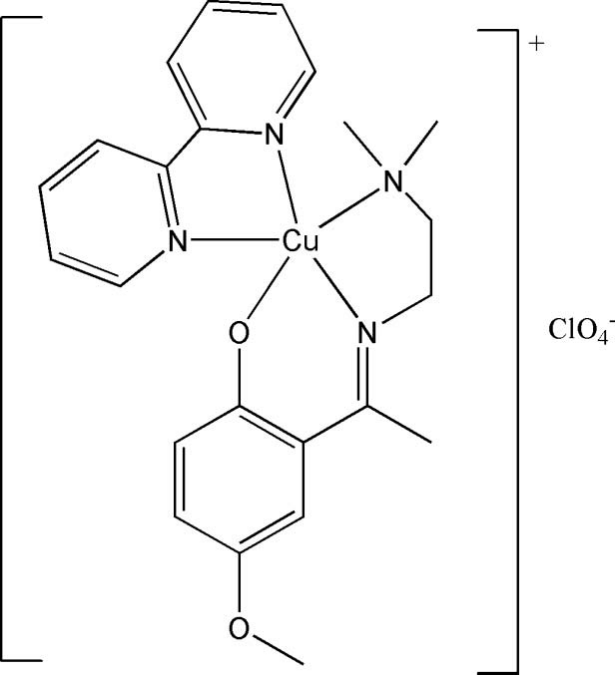


## Experimental

### 

#### Crystal data

[Cu(C_13_H_19_N_2_O_2_)(C_10_H_8_N_2_)]ClO_4_
*M* *_r_* = 554.49
Monoclinic, 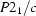

*a* = 10.1588 (10) Å
*b* = 18.2163 (17) Å
*c* = 13.3764 (13) Å
β = 92.610 (2)°
*V* = 2472.8 (4) Å^3^
*Z* = 4
Mo *K*α radiation
μ = 1.04 mm^−1^
*T* = 293 K
0.34 × 0.26 × 0.15 mm

#### Data collection

Bruker SMART 1000 CCD diffractometer
Absorption correction: multi-scan (*SADABS*; Sheldrick, 1996 ▶) *T*_min_ = 0.719, *T*_max_ = 0.860
13946 measured reflections
4859 independent reflections
3488 reflections with *I* > 2σ(*I*)
*R*_int_ = 0.037

#### Refinement

*R*[*F* ^2^ > 2σ(*F* ^2^)] = 0.038
*wR*(*F* ^2^) = 0.105
*S* = 0.98
4859 reflections
319 parameters
H-atom parameters constrained
Δρ_max_ = 0.33 e Å^−3^
Δρ_min_ = −0.30 e Å^−3^

### 

Data collection: *SMART* (Bruker, 1999 ▶); cell refinement: *SAINT* (Bruker, 1999 ▶); data reduction: *SAINT*; program(s) used to solve structure: *SHELXS97* (Sheldrick, 2008 ▶); program(s) used to refine structure: *SHELXL97* (Sheldrick, 2008 ▶); molecular graphics: *SHELXTL* (Sheldrick, 2008 ▶); software used to prepare material for publication: *SHELXTL*.

## Supplementary Material

Crystal structure: contains datablocks global, I. DOI: 10.1107/S1600536809014573/rk2135sup1.cif

Structure factors: contains datablocks I. DOI: 10.1107/S1600536809014573/rk2135Isup2.hkl

Additional supplementary materials:  crystallographic information; 3D view; checkCIF report

## Figures and Tables

**Fig. 1.**
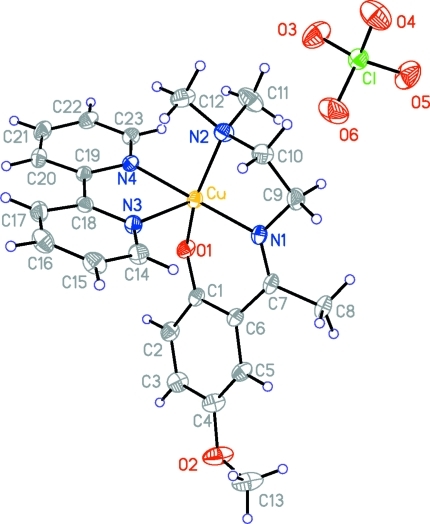
A view of the molecular structure with the atom numbering scheme. The displacement ellipsoids are shown at the 20% probability level. H atoms are presented as a small spheres of arbitrary radius.

## supplementary crystallographic information

### Comment

Though many bacteria convert CO_2_ into organic compounds by photosynthesis, utilization of CO_2_ as a chemical feedstock in industrial and laboratory is rare. Recently, reuse of CO_2_ has received great attention because of environmental concern. Polycarbonates (*PC*) have been wildly used in the modern chemical industry. Co–polymerization of CO_2_ with olefins may benefit from reducing the release of CO_2_ and generating potential industrial useful *PC*s. Therefore, there has been increasing interest in the development of efficient catalytic systems for the coupling of CO_2_ with heterocycles into polycarbonates (Inoue *et al.*, 1969). One of the major successes is the utilization of epoxides and CO_2_ as starting materials to prepare *PC*s and/or cyclic carbonates in the presence of a transition metal catalyst. Recently, Darensbourg *et al.*, (2001) disclosed the synthesis, characterization and catalytic studies of a series of *bis*–(salicylaldiminato)zinc complexes, in which the most active catalyst for co–polymerization of cyclohexene oxide and CO_2_ giving poly(cyclohexene carbonate) (>99% carbonate linkages, *Mn* = 41000 g^.^mol^-1^, *Mw*/*Mn* = 10.3) with a turnover frequency of 6.9 h^-1^. In addition, Shen *et al.* (2003) reported that binaphthyldiaminosalen–type Zn, Cu, and Co complexes efficiently catalyzed reactions of epoxides with CO_2_ to achieve five–membered ring cyclic carbonates in the presence of various catalytic amounts of organic bases. Noh *et al.*, (2007) disclosed catalytic studies of the binary system of [(*salen*)Co(III)complex] / (quaternary ammonium salt) for co–polymerization of propylene oxide and CO_2_. Most recently, a series of *N,N,O*–tridentate Schiff base zinc– and magnesium–complexes have been reported to be effective initiators / catalyst for *ROP* of lactide (Hung *et al.*, 2008; Hung & Lin, 2009). We report herein the synthesis and crystal structure of [*L*Cu(*bipy*)]ClO_4_, where *L* is title tridentate ligand and *bipy* is 2,2'–bipyridine, a potential catalyst for CO_2_ / epoxide coupling co–polymerization.

The solid structure of [*L*Cu(*bipy*)]^+^ ion reveals a monomeric Cu^II^ complex containing a six–member and a five–member ring coordinated from the tridentate salicylideneiminate ligand and a five–member ring coordinated from the bipyridine ligand. The geometry around Cu atom is penta–coordinated with a slight distorted square pyramidal environment in which all three of the *N,N,O*–tridentate donor atoms and one of the N atoms of the bipyridine lignad sitting on the equatorial plane, and another N atom of the bipyridine ligand at the axial position. The distances between the Cu atom and O1, N1, N2, N3 and N4 are 1.903 (2), 1.964 (2), 2.076 (2), 2.208 (2) and 2.044 (2) Å, respectively which are all within a normal distance for a Cu—O and Cu—N distance. These bond distances are similar to those found in other Schiff base Cu^II^ complexes (Dhar *et al.*, 2006).

### Experimental

The ligand, 2–{1–[2–(dimethylamino)ethylimino]ethyl}–4–methoxyphenol was prepared according to the method reported previously (Hung *et al.*, 2008). The title complex was synthesized by the following procedures: Cu(O*Ac*)_2_^.^H_2_O (0.197 g, 1.00 mmol) and 2,2'–bipyridine (0.199 g, 1.28 mmol) was stirred in *Et*OH (15 ml) at room temperature for 0.5 h. The 2–{1–[2–(dimethylamino)ethylimino]ethyl}–4–methoxyphenol (0.298 g, 1.0 mmol) in *Et*OH (10 ml) was added. The reaction mixture was then stirred for another 1 h, and an 10 ml ethanolic solution of NaClO_4_ (0.122 g, 1.0 mmol) was added producing green precipitate. The product was isolated by filtration and the resulting precipitate was crystallized from *Et*OH to yield green crystals.

### Refinement

The methyl H atoms were located and then constrained to an ideal geometry with C—H distances of 0.96 Å and *U*_iso_(H) = 1.5*U*_eq_(C), but each group was allowed to rotate freely about its C—C bond. All other H atoms were placed in geometrically idealized positions and constrained to ride on their parent atoms with C—H distances in the range 0.93 Å and 0.97 Å and *U*_iso_(H) = 1.2*U*_eq_(C).

### Crystal data

**Table:**

[Cu(C_13_H_19_N_2_O_2_)(C_10_H_8_N_2_)]ClO_4_	*F*(000) = 1148
*M**_r_* = 554.49	*D*_x_ = 1.489 Mg m^−^^3^
Monoclinic, *P*2_1_/*c*	Mo *K*α radiation, λ = 0.71073 Å
Hall symbol: -P 2ybc	Cell parameters from 4710 reflections
*a* = 10.1588 (10) Å	θ = 2.3–25.6°
*b* = 18.2163 (17) Å	µ = 1.04 mm^−^^1^
*c* = 13.3764 (13) Å	*T* = 293 K
β = 92.610 (2)°	Parallelpiped, green
*V* = 2472.8 (4) Å^3^	0.34 × 0.26 × 0.15 mm
*Z* = 4	

### Data collection

**Table:**

Bruker SMART 1000 CCD diffractometer	4859 independent reflections
Radiation source: fine–focus sealed tube	3488 reflections with *I* > 2σ(*I*)
graphite	*R*_int_ = 0.037
φ and ω scans	θ_max_ = 26.0°, θ_min_ = 1.9°
Absorption correction: multi-scan (*SADABS*; Sheldrick, 1996)	*h* = −12→12
*T*_min_ = 0.719, *T*_max_ = 0.860	*k* = −22→16
13946 measured reflections	*l* = −16→15

### Refinement

**Table:**

Refinement on *F*^2^	Primary atom site location: structure-invariant direct methods
Least-squares matrix: full	Secondary atom site location: difference Fourier map
*R*[*F*^2^ > 2σ(*F*^2^)] = 0.038	Hydrogen site location: inferred from neighbouring sites
*wR*(*F*^2^) = 0.105	H-atom parameters constrained
*S* = 0.98	*w* = 1/[σ^2^(*F*_o_^2^) + (0.06*P*)^2^] where *P* = (*F*_o_^2^ + 2*F*_c_^2^)/3
4859 reflections	(Δ/σ)_max_ = 0.001
319 parameters	Δρ_max_ = 0.33 e Å^−^^3^
0 restraints	Δρ_min_ = −0.30 e Å^−^^3^

### Special details

**Table:**

Geometry. All s.u.'s (except the s.u. in the dihedral angle between two l.s. planes) are estimated using the full covariance matrix. The cell s.u.'s are taken into account individually in the estimation of s.u.'s in distances, angles and torsion angles; correlations between s.u.'s in cell parameters are only used when they are defined by crystal symmetry. An approximate (isotropic) treatment of cell s.u.'s is used for estimating s.u.'s involving l.s. planes.
Refinement. Refinement of *F*^2^ against ALL reflections. The weighted *R*–factor *wR* and goodness of fit *S* are based on *F*^2^, conventional *R*–factors *R* are based on *F*, with *F* set to zero for negative *F*^2^. The threshold expression of *F*^2^ > σ(*F*^2^) is used only for calculating *R*–factors(gt) *etc*. and is not relevant to the choice of reflections for refinement. *R*–factors based on *F*^2^ are statistically about twice as large as those based on *F*, and *R*–factors based on ALL data will be even larger.

### Fractional atomic coordinates and isotropic or equivalent isotropic displacement parameters (Å^2^)

**Table:**

	*x*	*y*	*z*	*U*_iso_*/*U*_eq_	
Cu	0.62390 (3)	0.172001 (16)	0.82971 (2)	0.04095 (12)	
O1	0.6832 (2)	0.15346 (10)	0.96441 (14)	0.0532 (5)	
O2	0.9049 (3)	−0.10697 (14)	1.0997 (2)	0.1017 (10)	
N1	0.5494 (2)	0.07290 (12)	0.81586 (16)	0.0457 (5)	
N2	0.4979 (2)	0.19843 (13)	0.70841 (18)	0.0528 (6)	
N3	0.8094 (2)	0.16361 (12)	0.75071 (17)	0.0469 (6)	
N4	0.6989 (2)	0.27569 (11)	0.84447 (15)	0.0412 (5)	
C1	0.7293 (3)	0.08881 (15)	0.9916 (2)	0.0455 (6)	
C2	0.8227 (3)	0.08568 (17)	1.0724 (2)	0.0583 (8)	
H2A	0.8483	0.1293	1.1039	0.070*	
C3	0.8779 (4)	0.02114 (19)	1.1069 (2)	0.0681 (9)	
H3A	0.9395	0.0215	1.1605	0.082*	
C4	0.8411 (4)	−0.04469 (18)	1.0612 (2)	0.0665 (9)	
C5	0.7497 (3)	−0.04455 (16)	0.9842 (2)	0.0576 (8)	
H5A	0.7257	−0.0890	0.9544	0.069*	
C6	0.6895 (3)	0.02080 (15)	0.94751 (19)	0.0439 (6)	
C7	0.5849 (3)	0.01591 (15)	0.8687 (2)	0.0447 (7)	
C8	0.5192 (3)	−0.05759 (16)	0.8497 (2)	0.0605 (8)	
H8A	0.5193	−0.0850	0.9111	0.079 (10)*	
H8B	0.5666	−0.0844	0.8011	0.102 (13)*	
H8C	0.4300	−0.0500	0.8250	0.098 (13)*	
C9	0.4456 (3)	0.06785 (18)	0.7363 (2)	0.0629 (8)	
H9A	0.3599	0.0752	0.7639	0.075*	
H9B	0.4469	0.0197	0.7054	0.075*	
C10	0.4709 (3)	0.12658 (16)	0.6597 (2)	0.0603 (8)	
H10A	0.5457	0.1125	0.6214	0.072*	
H10B	0.3947	0.1309	0.6137	0.072*	
C11	0.3779 (4)	0.2319 (2)	0.7440 (3)	0.0901 (13)	
H11A	0.3197	0.2441	0.6879	0.135*	
H11B	0.4002	0.2758	0.7809	0.135*	
H11C	0.3349	0.1980	0.7867	0.135*	
C12	0.5537 (4)	0.24885 (18)	0.6336 (2)	0.0728 (10)	
H12A	0.4889	0.2579	0.5805	0.109*	
H12B	0.6301	0.2267	0.6066	0.109*	
H12C	0.5781	0.2944	0.6654	0.109*	
C13	0.8909 (5)	−0.1720 (2)	1.0445 (3)	0.1034 (15)	
H13A	0.9394	−0.2106	1.0783	0.155*	
H13B	0.9244	−0.1648	0.9792	0.155*	
H13C	0.7994	−0.1852	1.0380	0.155*	
C14	0.8536 (3)	0.10711 (16)	0.6971 (2)	0.0578 (8)	
H14A	0.8076	0.0630	0.6976	0.069*	
C15	0.9634 (3)	0.11167 (19)	0.6418 (2)	0.0630 (9)	
H15A	0.9903	0.0718	0.6045	0.076*	
C16	1.0329 (3)	0.1763 (2)	0.6427 (3)	0.0689 (9)	
H16A	1.1076	0.1809	0.6055	0.083*	
C17	0.9908 (3)	0.23447 (17)	0.6995 (2)	0.0572 (8)	
H17A	1.0379	0.2783	0.7021	0.069*	
C18	0.8784 (3)	0.22671 (14)	0.75206 (19)	0.0415 (6)	
C19	0.8210 (3)	0.28739 (14)	0.81221 (18)	0.0402 (6)	
C20	0.8879 (3)	0.35225 (16)	0.8336 (2)	0.0531 (7)	
H20A	0.9728	0.3592	0.8123	0.064*	
C21	0.8265 (3)	0.40656 (17)	0.8872 (2)	0.0611 (8)	
H21A	0.8702	0.4503	0.9025	0.073*	
C22	0.7019 (3)	0.39554 (15)	0.9173 (2)	0.0546 (8)	
H22A	0.6587	0.4319	0.9520	0.066*	
C23	0.6409 (3)	0.32963 (14)	0.8954 (2)	0.0480 (7)	
H23A	0.5561	0.3220	0.9166	0.058*	
Cl	0.25347 (7)	0.09143 (4)	0.40216 (5)	0.05096 (19)	
O3	0.2755 (3)	0.16261 (12)	0.4419 (2)	0.0957 (9)	
O4	0.2085 (3)	0.09639 (16)	0.30025 (18)	0.0956 (9)	
O5	0.1576 (3)	0.05517 (15)	0.4574 (2)	0.0958 (8)	
O6	0.3729 (2)	0.05090 (16)	0.4085 (2)	0.0970 (9)	

### Atomic displacement parameters (Å^2^)

**Table:**

	*U*^11^	*U*^22^	*U*^33^	*U*^12^	*U*^13^	*U*^23^
Cu	0.0450 (2)	0.03477 (19)	0.04338 (19)	−0.00436 (14)	0.00530 (14)	−0.00633 (13)
O1	0.0719 (14)	0.0383 (11)	0.0488 (11)	−0.0068 (9)	−0.0037 (10)	−0.0082 (8)
O2	0.157 (3)	0.0660 (17)	0.0788 (17)	0.0307 (17)	−0.0360 (18)	0.0011 (13)
N1	0.0480 (14)	0.0429 (13)	0.0461 (12)	−0.0095 (11)	0.0028 (10)	−0.0070 (11)
N2	0.0570 (15)	0.0454 (14)	0.0554 (14)	0.0058 (12)	−0.0036 (12)	−0.0100 (11)
N3	0.0431 (13)	0.0430 (14)	0.0552 (14)	0.0014 (10)	0.0088 (11)	−0.0067 (10)
N4	0.0466 (13)	0.0356 (12)	0.0416 (12)	−0.0007 (10)	0.0033 (10)	−0.0018 (9)
C1	0.0533 (17)	0.0415 (16)	0.0424 (14)	−0.0072 (13)	0.0103 (12)	−0.0023 (12)
C2	0.071 (2)	0.0517 (19)	0.0519 (17)	−0.0082 (16)	−0.0031 (15)	−0.0078 (14)
C3	0.082 (2)	0.070 (2)	0.0515 (18)	−0.0007 (19)	−0.0059 (17)	−0.0052 (16)
C4	0.089 (3)	0.057 (2)	0.0532 (18)	0.0107 (18)	0.0023 (18)	−0.0003 (15)
C5	0.080 (2)	0.0413 (17)	0.0515 (17)	−0.0015 (15)	0.0065 (16)	−0.0040 (13)
C6	0.0526 (16)	0.0415 (15)	0.0383 (14)	−0.0049 (13)	0.0089 (12)	−0.0015 (11)
C7	0.0517 (16)	0.0389 (15)	0.0451 (15)	−0.0108 (13)	0.0191 (13)	−0.0092 (12)
C8	0.074 (2)	0.0480 (18)	0.0608 (19)	−0.0214 (16)	0.0146 (17)	−0.0062 (15)
C9	0.061 (2)	0.060 (2)	0.066 (2)	−0.0155 (16)	−0.0089 (16)	−0.0077 (16)
C10	0.070 (2)	0.0530 (19)	0.0569 (18)	0.0031 (16)	−0.0116 (16)	−0.0116 (14)
C11	0.063 (2)	0.103 (3)	0.103 (3)	0.028 (2)	−0.010 (2)	−0.035 (2)
C12	0.099 (3)	0.055 (2)	0.063 (2)	0.0052 (19)	−0.0126 (19)	0.0072 (16)
C13	0.149 (4)	0.068 (3)	0.092 (3)	0.037 (3)	−0.011 (3)	0.005 (2)
C14	0.0549 (18)	0.0486 (18)	0.070 (2)	0.0049 (14)	0.0082 (15)	−0.0145 (15)
C15	0.060 (2)	0.066 (2)	0.063 (2)	0.0147 (17)	0.0092 (16)	−0.0190 (16)
C16	0.0503 (19)	0.091 (3)	0.067 (2)	0.0089 (18)	0.0195 (16)	−0.0087 (18)
C17	0.0470 (17)	0.059 (2)	0.0658 (19)	−0.0041 (14)	0.0093 (15)	0.0006 (15)
C18	0.0399 (14)	0.0445 (15)	0.0398 (14)	0.0019 (12)	−0.0011 (11)	0.0024 (11)
C19	0.0429 (15)	0.0402 (15)	0.0371 (13)	−0.0018 (12)	−0.0026 (11)	0.0038 (11)
C20	0.0515 (18)	0.0513 (18)	0.0566 (17)	−0.0134 (14)	0.0023 (14)	−0.0014 (14)
C21	0.076 (2)	0.0430 (17)	0.0639 (19)	−0.0151 (16)	−0.0035 (17)	−0.0052 (14)
C22	0.077 (2)	0.0381 (16)	0.0488 (17)	0.0009 (15)	0.0025 (15)	−0.0054 (12)
C23	0.0541 (17)	0.0423 (16)	0.0480 (16)	0.0021 (13)	0.0060 (13)	−0.0046 (12)
Cl	0.0475 (4)	0.0508 (4)	0.0552 (4)	0.0010 (3)	0.0103 (3)	0.0000 (3)
O3	0.138 (3)	0.0527 (15)	0.097 (2)	−0.0073 (15)	0.0050 (18)	−0.0065 (13)
O4	0.0810 (17)	0.146 (3)	0.0593 (15)	0.0118 (17)	−0.0038 (13)	−0.0072 (15)
O5	0.0868 (18)	0.0864 (19)	0.118 (2)	−0.0077 (15)	0.0486 (16)	0.0223 (15)
O6	0.0599 (15)	0.113 (2)	0.118 (2)	0.0323 (15)	0.0115 (14)	0.0019 (17)

### Geometric parameters (Å, °)

**Table:**

Cu—O1	1.9037 (19)	C9—H9B	0.9700
Cu—N1	1.963 (2)	C10—H10A	0.9700
Cu—N4	2.043 (2)	C10—H10B	0.9700
Cu—N2	2.077 (2)	C11—H11A	0.9600
Cu—N3	2.207 (2)	C11—H11B	0.9600
O1—C1	1.313 (3)	C11—H11C	0.9600
O2—C4	1.393 (4)	C12—H12A	0.9600
O2—C13	1.400 (4)	C12—H12B	0.9600
N1—C7	1.298 (3)	C12—H12C	0.9600
N1—C9	1.466 (3)	C13—H13A	0.9600
N2—C11	1.462 (4)	C13—H13B	0.9600
N2—C10	1.482 (4)	C13—H13C	0.9600
N2—C12	1.490 (4)	C14—C15	1.369 (4)
N3—C14	1.343 (3)	C14—H14A	0.9300
N3—C18	1.346 (3)	C15—C16	1.372 (4)
N4—C19	1.348 (3)	C15—H15A	0.9300
N4—C23	1.347 (3)	C16—C17	1.383 (4)
C1—C2	1.407 (4)	C16—H16A	0.9300
C1—C6	1.423 (4)	C17—C18	1.375 (4)
C2—C3	1.373 (4)	C17—H17A	0.9300
C2—H2A	0.9300	C18—C19	1.501 (4)
C3—C4	1.389 (4)	C19—C20	1.386 (4)
C3—H3A	0.9300	C20—C21	1.387 (4)
C4—C5	1.355 (4)	C20—H20A	0.9300
C5—C6	1.416 (4)	C21—C22	1.360 (4)
C5—H5A	0.9300	C21—H21A	0.9300
C6—C7	1.465 (4)	C22—C23	1.376 (4)
C7—C8	1.512 (4)	C22—H22A	0.9300
C8—H8A	0.9600	C23—H23A	0.9300
C8—H8B	0.9600	Cl—O5	1.413 (2)
C8—H8C	0.9600	Cl—O3	1.416 (2)
C9—C10	1.512 (4)	Cl—O6	1.419 (2)
C9—H9A	0.9700	Cl—O4	1.421 (2)
			
O1—Cu—N1	91.73 (9)	N2—C10—C9	111.1 (3)
O1—Cu—N4	88.40 (8)	N2—C10—H10A	109.4
N1—Cu—N4	179.21 (9)	C9—C10—H10A	109.4
O1—Cu—N2	159.65 (9)	N2—C10—H10B	109.4
N1—Cu—N2	85.30 (9)	C9—C10—H10B	109.4
N4—Cu—N2	94.31 (9)	H10A—C10—H10B	108.0
O1—Cu—N3	101.58 (9)	N2—C11—H11A	109.5
N1—Cu—N3	103.01 (9)	N2—C11—H11B	109.5
N4—Cu—N3	77.72 (8)	H11A—C11—H11B	109.5
N2—Cu—N3	98.71 (9)	N2—C11—H11C	109.5
C1—O1—Cu	121.01 (16)	H11A—C11—H11C	109.5
C4—O2—C13	117.4 (3)	H11B—C11—H11C	109.5
C7—N1—C9	121.2 (2)	N2—C12—H12A	109.5
C7—N1—Cu	125.99 (18)	N2—C12—H12B	109.5
C9—N1—Cu	112.81 (18)	H12A—C12—H12B	109.5
C11—N2—C10	111.8 (3)	N2—C12—H12C	109.5
C11—N2—C12	108.1 (3)	H12A—C12—H12C	109.5
C10—N2—C12	108.5 (2)	H12B—C12—H12C	109.5
C11—N2—Cu	109.6 (2)	O2—C13—H13A	109.5
C10—N2—Cu	103.51 (18)	O2—C13—H13B	109.5
C12—N2—Cu	115.31 (19)	H13A—C13—H13B	109.5
C14—N3—C18	118.4 (2)	O2—C13—H13C	109.5
C14—N3—Cu	128.5 (2)	H13A—C13—H13C	109.5
C18—N3—Cu	112.95 (17)	H13B—C13—H13C	109.5
C19—N4—C23	118.5 (2)	N3—C14—C15	122.8 (3)
C19—N4—Cu	117.37 (17)	N3—C14—H14A	118.6
C23—N4—Cu	123.61 (19)	C15—C14—H14A	118.6
O1—C1—C2	118.0 (2)	C14—C15—C16	118.6 (3)
O1—C1—C6	125.1 (3)	C14—C15—H15A	120.7
C2—C1—C6	116.9 (3)	C16—C15—H15A	120.7
C3—C2—C1	122.9 (3)	C15—C16—C17	119.3 (3)
C3—C2—H2A	118.5	C15—C16—H16A	120.3
C1—C2—H2A	118.5	C17—C16—H16A	120.3
C2—C3—C4	119.6 (3)	C18—C17—C16	119.1 (3)
C2—C3—H3A	120.2	C18—C17—H17A	120.4
C4—C3—H3A	120.2	C16—C17—H17A	120.4
C5—C4—C3	119.6 (3)	N3—C18—C17	121.7 (2)
C5—C4—O2	125.0 (3)	N3—C18—C19	114.9 (2)
C3—C4—O2	115.4 (3)	C17—C18—C19	123.4 (3)
C4—C5—C6	122.4 (3)	N4—C19—C20	121.2 (2)
C4—C5—H5A	118.8	N4—C19—C18	116.2 (2)
C6—C5—H5A	118.8	C20—C19—C18	122.6 (2)
C5—C6—C1	118.5 (3)	C21—C20—C19	119.1 (3)
C5—C6—C7	119.1 (2)	C21—C20—H20A	120.4
C1—C6—C7	122.3 (2)	C19—C20—H20A	120.4
N1—C7—C6	121.2 (2)	C22—C21—C20	119.6 (3)
N1—C7—C8	120.5 (3)	C22—C21—H21A	120.2
C6—C7—C8	118.3 (3)	C20—C21—H21A	120.2
C7—C8—H8A	109.5	C21—C22—C23	118.8 (3)
C7—C8—H8B	109.5	C21—C22—H22A	120.6
H8A—C8—H8B	109.5	C23—C22—H22A	120.6
C7—C8—H8C	109.5	N4—C23—C22	122.8 (3)
H8A—C8—H8C	109.5	N4—C23—H23A	118.6
H8B—C8—H8C	109.5	C22—C23—H23A	118.6
N1—C9—C10	108.0 (2)	O5—Cl—O3	109.42 (18)
N1—C9—H9A	110.1	O5—Cl—O6	109.47 (17)
C10—C9—H9A	110.1	O3—Cl—O6	109.50 (18)
N1—C9—H9B	110.1	O5—Cl—O4	109.41 (17)
C10—C9—H9B	110.1	O3—Cl—O4	109.93 (18)
H9A—C9—H9B	108.4	O6—Cl—O4	109.10 (17)
